# Application of machine learning methods in prediction of the body constitution types and transformation trends of traditional Chinese medicine: from the datasets of questionnaire survey on elderly people in Southwest China

**DOI:** 10.3389/fmed.2026.1698576

**Published:** 2026-01-15

**Authors:** Yuzhi Huo, Jia Wang, Yingchun He, Yang Zhao, Fei Wang, Mei Zhang

**Affiliations:** 1 School of Clinical Medicine, Chengdu University of Traditional Chinese Medicine, Chengdu, China; 2 Department of Traditional Chinese Medicine, The Third People’s Hospital of Chengdu, Chengdu, China; 3 College of Basic Medical Sciences, Dalian Medical University, Dalian, China; 4 School of Chinese Medicine, Sichuan College of Traditional Chinese Medicine, Mianyang, China; 5 School of Management, Chengdu University of Traditional Chinese Medicine, Chengdu, China

**Keywords:** dynamic transformation, elderly, machine learning, predictive modeling, TCM constitution

## Abstract

**Background:**

Traditional Chinese Medicine (TCM) constitution theory posits that constitution is dynamic, yet most research is cross-sectional. This study aims to bridge this gap by developing machine learning models using longitudinal cohort data to predict dynamic constitutional changes in the elderly. The objectives were twofold: to predict an individual’s specific constitution type at a future time point and to classify the transformation trend between assessments.

**Methods:**

This study utilized a large-scale longitudinal cohort, including 54,990 records from the TCM Elderly Constitution Questionnaire (TCMECQ) for model development and 2,181 records for external validation. Five machine learning models, including Support Vector Machine (SVM), Random Forest Classifier (RFC), Decision Tree (DT), K-Nearest Neighbors (KNN), and Neural Network (NN), were trained and validated. Model training and evaluation were performed using 10-fold cross-validation to ensure robustness. We defined two predictive outcomes: the specific future constitution type (nine-class) and the transformation trend (“better,” “indeterminate,” or “worse”), classified based on expert consensus. We also evaluated the predictive performance of 13 common biochemical indicators.

**Results:**

For predicting the specific future constitution type, SVM demonstrated the best performance with an internal validation accuracy of 99.47%. In the core task of classifying the transformation trend, the RFC model was superior, achieving an accuracy of 96.92% (Kappa = 0.9505). However, all models performed poorly when predicting the “worse” trend in external validation. Analyses using biochemical indicators showed moderate performance for identifying “better” (accuracy >80%) and “indeterminate” (accuracy >90%) states but failed to predict the “worse” trend (accuracy <5%).

**Conclusion:**

This study establishes the first machine learning framework to effectively predict the dynamic evolution of TCM constitution. Our findings show that SVM excels at predicting specific future states, while RFC is more adept at capturing overall evolutionary trends. This framework provides a novel quantitative tool for advancing proactive health management aligned with the TCM principle of “Zhi Wei Bing”. The models’ inability to reliably predict constitutional deterioration highlights the critical need to incorporate multi-modal data to capture complex pathophysiological mechanisms in future research.

## 1 Introduction

Traditional Chinese Medicine (TCM), rooted in a holistic philosophy that emphasizes the harmony between humanity and nature (“Tian Ren He Yi”), views body constitution as a comprehensive and relatively stable manifestation of an individual’s life processes (1). This constitutional state, which integrates morphological structure, physiological function, and psychological condition, is a cornerstone of personalized healthcare in TCM, guiding the principles of “treatment based on constitution” (Bian Ti Shi Zhi) and preventive medicine (“Zhi Wei Bing”) (2). In the contemporary era of big data, the convergence of this ancient wisdom with modern artificial intelligence (AI) has opened new frontiers for the objective and large-scale assessment of TCM constitution.

The application of machine learning (ML) and deep learning (DL) has yielded various models for constitution recognition, primarily utilizing physiological signals such as speech, facial images, and pulse waves (3–6). While these approaches have demonstrated technical feasibility, they often function as “black boxes”, presenting a significant challenge for clinical interpretation and validation by TCM practitioners. A primary limitation is their frequent detachment from the foundational theories and diagnostic knowledge of TCM, making it difficult to understand the rationale behind their outputs (7). In contrast, the Traditional Chinese Medicine Elderly Constitution Questionnaire (TCMECQ), established based on the national standard and developed by Professor Wang Qi and his team, is deeply embedded in TCM theory and the four diagnostic methods (8, 9). Its widespread application, particularly within China’s National Basic Public Health Service program for the elderly, has generated an unprecedented repository of longitudinal data, offering a superior foundation for developing more accurate and, crucially, more interpretable AI models for constitution analysis (10). Recent international studies have explored a broad range of algorithms for biomedical signal-based diagnosis, including Adaboost-based ensemble learning (11), wavelet-domain correlation analysis (12), and Extreme Learning Machines (13) for emotional or cognitive dysfunction classification. These approaches emphasize not only classification accuracy but also model interpretability and cross-population generalizability. Compared with deep learning architectures that often rely on abstract, high-dimensional representations of raw signals, the present study focuses on five mainstream classifiers (SVM, RFC, DT, NN, and KNN) to establish a baseline framework. These methods offer a distinct advantage in interpretability: they utilize explicit, clinically defined symptoms from the TCMECQ as input features rather than opaque pixel or wave data, ensuring that the semantic link between specific symptom clusters and constitution types remains traceable (e.g., via decision paths in DT or feature importance in RFC).

However, a more profound research gap lies not merely in the static identification of constitution but in understanding its dynamic nature. A central tenet of TCM constitutional theory, as articulated by Professor Wang Qi, is “dynamic variability” (Ti Zhi Ke Bian Lun), which posits that an individual’s constitution is not immutable but evolves over time in response to age, lifestyle, environment, and health status (14). While the precise mechanisms of this theory are a subject of academic exploration, our study is guided by it to empirically model an observed phenomenon. To date, the vast majority of studies have been cross-sectional, focusing only on the static distribution of constitution types at a single point in time. Our own 7-year longitudinal cohort observationally confirmed this phenomenon, noting that individuals do change constitution types over time. The longitudinal transformation laws, predictive factors, and underlying patterns of constitutional change remain a largely unexplored frontier. This oversight is significant, as the syndrome data inherent in constitutional assessment is characterized by nonlinearity, spatiotemporal dynamics, and multi-level complexity, making it an ideal challenge for advanced computational analysis (15).

The foundation of this study is a large-scale, longitudinal dataset derived from the TCMECQ (9, 10). Originating from China’s National Basic Public Health Service program, this dataset systematically tracked 54,990 elderly individuals from 2017 to 2024. Its significance extends beyond mere scale; it provides the essential temporal dimension required to operationalize the TCM principle of “Zhi Wei Bing” (preventive treatment of disease). “Zhi Wei Bing” emphasizes intervening before pathology manifests (2), aligning closely with modern proactive health management. However, implementing this requires the ability to foresee health trajectories. By leveraging this unique longitudinal data, we can move beyond static snapshots to empirically model dynamic constitutional changes, thereby providing a scientific, predictive tool to guide early intervention and realize the goal of disease prevention.

Therefore, this study aims to address this critical gap by leveraging a large-scale, longitudinal dataset of elderly individuals derived from the TCMECQ. Our primary objective is to construct and validate ML models capable of not only accurately identifying an individual’s current body constitution but also predicting its future transformation trajectory. Furthermore, we will investigate the influence of key physiological and biochemical indicators on these constitutional shifts, seeking to bridge TCM diagnostics with modern clinical data. By elucidating the dynamic patterns of body constitution, this research holds significant value. It promises to provide a novel, quantitative paradigm for proactive health management and offer scientific evidence for the modernization of the TCM “Zhi Wei Bing” concept. From a health economics perspective, trajectory-aware predictions based on TCMECQ can inform the economic value of medication decisions (e.g., continuation, escalation, de-prescribing, or watchful waiting). This enables more cost-effective, timely, and individualized interventions, helping to reduce avoidable expenditures for patients, improve the efficiency of publicly funded services, and ultimately lessen the overall healthcare economic burden.

## 2 Methods

### 2.1 Study design and patient selection

**Figure 1:**
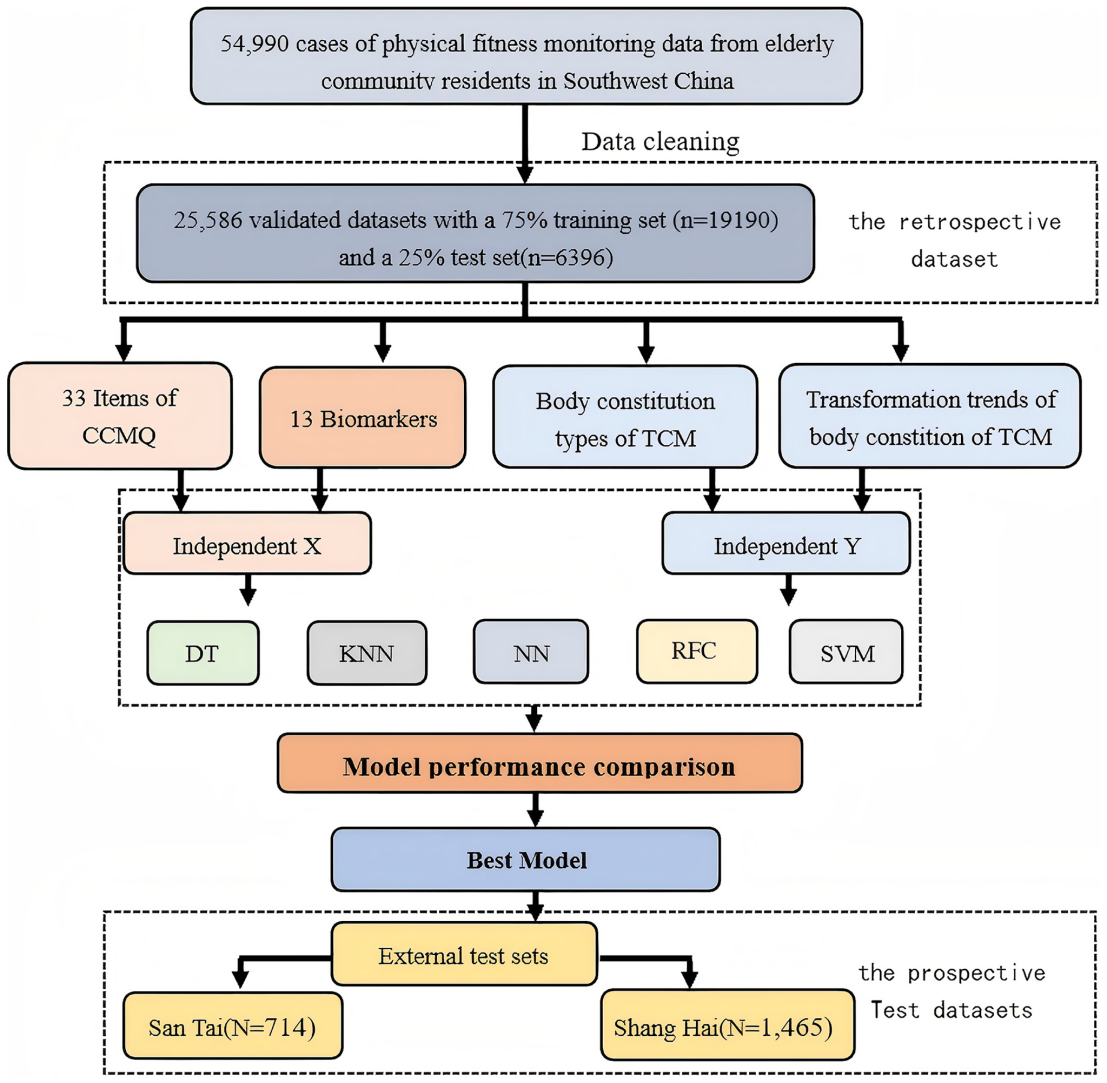
Overview of the data acquisition, modeling, and model performance evaluation. TCM, Traditional Chinese Medicine; DT, Decision Tree, KNN, K-Nearest Neighbors; NN, Neural Network; RFC, Random Forest Classifier; SVM, Support Vector Machine.

This study was approved by the Medical Ethics Committee of the Affiliated Hospital of Chengdu University of Traditional Chinese Medicine (Ethics Approval No. 2021KL-055A). All participants provided written informed consent. The patient data were included in two datasets: a internal dataset for model development and a external test dataset. The internal dataset consists of TCM constitution monitoring data collected from the Deyang Public Health Center between 2017 and 2024. Data acquisition followed a census-like total population sampling strategy rather than random sampling. We included the consecutive health records of all registered elderly individuals (aged 60 and above) who participated in the National Basic Public Health Service program during this period, covering a total of 54,990 records from elderly individuals aged 60 and above. The external test dataset includes TCM constitution monitoring data from July 2023 to October 2024, collected from nursing homes or community health centers in Shanghai and Santai. Specifically, 1,467 records were collected from Shanghai and 714 records from Santai. The cohort comprised elderly individuals who underwent TCMECQ assessments. The exclusion criteria were as follows: (1) Unknown TCM constitution type; (2) Incomplete demographic or lifestyle data; (3) Unknown survival information (Figure 1).

### 2.2 Observational indicators

#### 2.2.1 Body constitution type of TCM and transformation of TCM

According to the TCMECQ, the nine constitutional types-Balanced constitution (BC), Qi-deficiency constitution (QDC), Yang-deficiency constitution (YaDC), Yin-deficiency constitution (YiDC), Phlegm-dampness constitution (PDC), Damp-heat constitution (DHC), Blood-stasis constitution (BSC), Qi-stagnation constitution (QSC), and Inherited-special constitution (ISC)-are classified using the nine-part method of TCM in strict accordance with the national standard Classification and Determination of Constitution in Traditional Chinese Medicine (ZYYXH/T157-2009) (8), detail in Supplementary Table 1.

**Figure 2:**
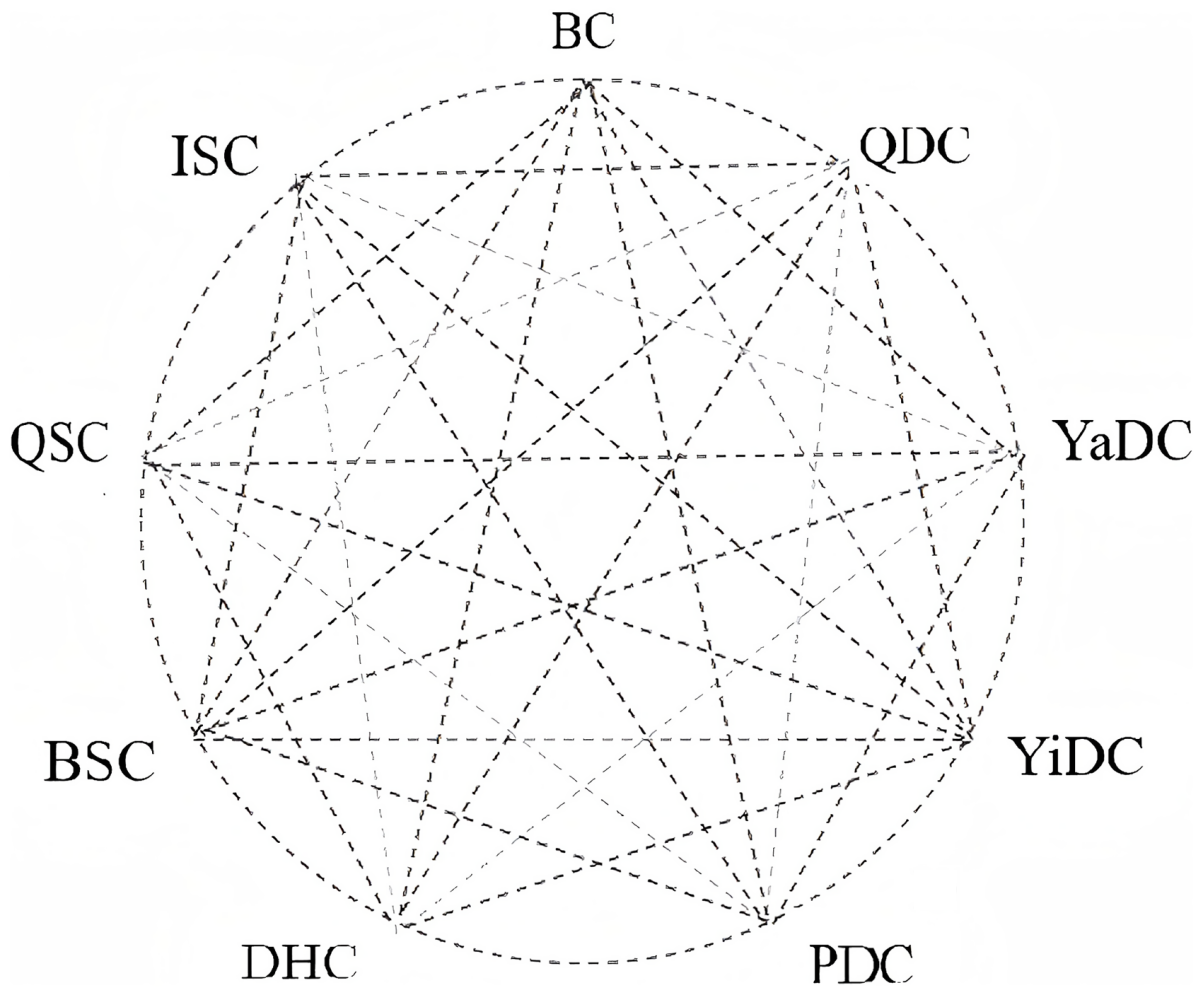
9 basic types of body constitution and transformation relationships. BC, Balanced constitution; QDC, Qi-deficiency constitution; YaDC, Yang-deficiency constitution; YiDC, Yin-deficiency constitution; PDC, Phlegm-dampness constitution; DHC, Damp-heat constitution; BSC, Blood-stasis constitution; QSC, Qi-stagnation constitution; ISC, Inherited-special constitution.

Building on this classification, body constitution transformation is defined as the ordered change in the constitution identification result between two adjacent TCMECQ assessments, denoted A → B, where A and B are any of the nine types. When A = B the event is labeled stability; when A ≠ B it is labeled change. In total, there are 9 × 9 = 81 possible ordered events, including 72 changes and 9 stability events, as illustrated in Figure 2. This definition reframes constitution from a one-off static label into a time-evolving, observable state–transition process, amenable to counting and modeling at both individual and population levels.

We selected body constitution type of TCM as the outcome because it aligns with the doctrinal view of constitutional variability and the operational logic of proactive management. Dynamic evidence on whether, where, and how often state switches occur directly informs follow-up schedules, intervention timing, and resource allocation-evidence that a single static label cannot provide. It also enhances clinical interpretability, since categorical state transitions are more actionable for TCM decision-making than small oscillations in scores. Methodologically and from a health-economics perspective, transitions naturally fit state-transition and time-series modeling (e.g., Markov frameworks), enabling estimation of transition probabilities, dwell times, and intervention effects that can feed into cost–effectiveness analyses.

We did not use changes in TCMECQ scores as the outcome due to measurement and interpretability constraints. The nine subscale scores are not on a common metric, so cross-dimension differences are not inherently comparable, and there is no widely validated minimal clinically important difference (MCID) to anchor the clinical meaning of score changes. Near-threshold variability and ceiling/floor effects-particularly in older adults-raise the risk of mistaking measurement noise for true change. Most importantly, small score shifts rarely map directly onto treatment adjustments or management pathways, whereas categorical state transitions do. For these reasons, we pre-specified ordered constitution transformations (including stability) as Outcome Indicator 1 to maximize clinical interpretability and methodological robustness and to provide a solid basis for subsequent dynamic modeling and health-economic evaluation.

#### 2.2.2 Transformation trend of body constitution of TCM

For the primary predictive modeling, the core outcome variable was further operationalized as a derived, three-class indicator of directional constitution change, categorized as “better,” “worse,” or “indeterminate”. This classification was chosen not merely on the basis of raw type-to-type transitions between different constitution types, but by further summarizing and categorizing them, primarily based on key considerations such as enhancing clinical interpretability and improving model robustness. First, this framework aligns with clinical reasoning. TCM intervention is goal-oriented, with the BC as the ideal state; therefore, the directional information of whether an individual is moving toward or away from this target is more clinically meaningful than the specific identity of the transition itself. Second, it acknowledges the non-linear nature of TCM constitution theory. The nine constitution types do not form a simple linear hierarchy of desirability in the elderly, and the relative merit of transitions between certain pairs is ambiguous or context-dependent. The “indeterminate” category formally accommodates this clinical uncertainty, thereby avoiding the introduction of label noise into the model. Finally, this indicator has direct relevance for health economic evaluation. A model that predicts a trend of “better” or “worse” provides direct evidence for assessing the cost-effectiveness of interventions, such as medication, thus guiding more efficient and effective health management strategies. The “indeterminate” category formally accommodates this clinical uncertainty, thereby avoiding the introduction of label noise into the model.

The criteria for assigning each transition to one of the three classes were established through a rigorous, multi-stage expert consensus process (Delphi method). Given the exploratory nature of quantifying dynamic constitution trends, we relied on this consensus to ensure the clinical validity of the labels. We invited 12 nationally recognized leading experts in the field of TCM constitution to participate. A structured expert questionnaire was designed, covering all 81 possible constitution transition events (including the stability of each of the nine types). The experts provided their judgments on the directionality of each transition based on their theoretical knowledge and extensive clinical experience (Supplementary Table 3). After collecting the responses, we coded and aggregated the judgments and applied statistical methods, including consistency testing, to assess the level of agreement. Crucially, items with significant disagreement (e.g., a mix of “1,” “0,” and “−1” votes) or inherent ambiguity (such as all 9 “stability” A → A transitions) were not classified mechanically. Instead, these items were subjected to further rounds of review and structured discussion until a final consensus was reached. The “indeterminate” category was the key methodological strategy to resolve ambiguity, particularly for A → A transitions where the direction of change is unknown from the label alone. The resulting standardized mapping scheme, which provides an authoritative and consistent basis for our outcome variable, is detailed in Supplementary Tables 2, 3.

### 2.3 Data analysis

The predictive power of TCM constitution type and transformation trend were assessed using five machine learning models: Random Forest Classifier (RFC), Support Vector Machine (SVM), Decision Tree (DT), Neural Network (NN), and K-Nearest Neighbors Classifier (KNN). The research dataset includes an internal validation set (*N* = 25,586) and two external validation sets. The two external validation sets are the Shanghai cohort (*N* = 1,465) and the Santai cohort (*N* = 714), The internal cohort dataset was divided into a training set (75% of the total data) and a test set (25% of the total data) to facilitate model training and performance evaluation. Additionally, three external test datasets were utilized to evaluate the external applicability of the models. To evaluate the performance of the model, 10-fold cross-validation was employed.

#### 2.3.1 Data preprocessing

A standardized data preprocessing pipeline was implemented prior to model construction to ensure data quality, minimize bias introduced by missing values and outliers, and improve model stability and generalizability. First, data completeness was assessed at both the feature and sample levels. Features with more than 20% missing values were excluded from subsequent analysis. For samples with sporadic missing values (<20% per variable), missing entries were imputed using mean imputation based on the corresponding feature distribution in the training cohort only, in order to avoid information leakage. Second, all continuous variables were normalized using Z-score standardization, defined as (x − *μ*) / *σ*, where μ and σ denote the mean and standard deviation estimated from the training data. The same normalization parameters were subsequently applied to the internal test set and both external validation cohorts to ensure preprocessing consistency. Third, outlier detection was performed using the interquartile range (IQR) method. Observations falling outside the range of [Q1–1.5 × IQR, Q3 + 1.5 × IQR] were considered outliers. For biologically implausible extreme values, outlier samples were removed; for moderate deviations, values were winsorized to the corresponding boundary. Finally, all preprocessing steps—including missing value handling, normalization, and outlier processing—were conducted exclusively within the training dataset, and the learned preprocessing parameters were strictly transferred to the validation and external test datasets to prevent data leakage.

#### 2.3.2 Feature selection

The feature selection was driven by the TCM constitution assessment results acquired through the CCM scale. The converted scores derived from the nine TCM constitution classifications served as pivotal features in our models.

#### 2.3.3 Model optimization and hyperparameter tuning

To fine-tune the hyperparameters of five models-RFC, DT, SVM, KNN, and NN- a comprehensive Grid Search algorithm was meticulously employed. For instance, in RFC, the number of trees (ntree) was tested in the range of 100–500, and the number of features (mtry) in {2, 5, 10}. For SVM, the kernel type (linear, polynomial, RBF) and the regularization parameter C were tuned across {0.1, 1, 10}. For KNN, the neighborhood size k ranged from 3 to 15, and for NN, hidden layer sizes varied between 64 and 256 neurons. All parameter sets were evaluated via 10-fold cross-validation, with accuracy and Kappa values used as the selection criteria. This approach systematically evaluated a predefined range of parameter values to identify the optimal combination that maximizes model performance. Specifically, for the RFC model, the hyperparameter mtry (the number of variables randomly sampled as candidates at each split) was tested with values of (2, 5, 10).

This study was conducted using R version 4.4.0 (2024-04-24 ucrt) on a Windows 10 ×64 platform, with random number generation based on the Mersenne-Twister algorithm. Several core R packages, such as “stats,” “graphics,” “base,” “readxl” and “caret,” were used throughout the study. These packages provided essential support for data processing and analysis.

#### 2.3.4 Model performance evaluation

The experimental results were evaluated and presented using a confusion matrix. Several key metrics were employed to assess model performance, including Accuracy, Sensitivity, Specificity, Positive Predictive Value (PPV), and Negative Predictive Value (NPV). The experiment was conducted using 10-fold cross-validation to ensure the reliability and generalizability of the results.

Accuracy represents the proportion of correctly predicted samples out of the total samples and is calculated as (Equation 1):


$$\frac{\mathrm{TP}+\mathrm{TN}}{\mathrm{TP}+\mathrm{TN}+\mathrm{FP}+\mathrm{FN}}$$ (1)

where TP represents the number of true positives, FP represents false positives, TN represents true negatives, and FN represents false negatives.

Sensitivity (also known as the true positive rate) reflects the proportion of actual samples of a given constitution type that the model correctly identifies. It is calculated as (Equation 2):


$$\frac{\mathrm{TP}}{\mathrm{TP}+\mathrm{FN}}$$ (2)

Specificity (also known as the true negative rate) reflects the proportion of samples not of a given constitution type that are correctly identified by the model. It is calculated as (Equation 3):


$$\frac{\mathrm{TN}}{\mathrm{TN}+\mathrm{FP}}$$ (3)

Positive Predictive Value (PPV) represents the proportion of samples predicted as a certain constitution type that are actually of that type. It is calculated as (Equation 4):


$$\frac{\mathrm{TP}}{\mathrm{TP}+\mathrm{FP}}$$ (4)

Negative Predictive Value (NPV) represents the proportion of samples predicted as not being a certain constitution type that are actually not of that type. It is calculated as (Equation 5):


$$\frac{\mathrm{TN}}{\mathrm{TN}+\mathrm{FN}}$$ (5)

## 3 Results

### 3.1 Predictive performance of machine learning models for constitution type classification

In this section, we evaluated the overall performance of five machine learning models (SVM, RFC, KNN, NN, and DT) in predicting TCM constitution types using 33 measurement items.

#### 3.1.1 Overall model performance

In the internal validation dataset (*N* = 25,586), SVM demonstrated the best overall predictive performance, with an accuracy of 99.47% (95% CI: 99.26–99.63) and a Kappa value of 0.991. The RFC model followed with an accuracy of 95.48% (95% CI: 94.94–95.98), although its average sensitivity was lower (56.67%). In contrast, the KNN, NN, and DT models showed moderate performance; despite high specificities (>95%), their accuracies were below 90%, and sensitivities were poor (<30%). The superiority of SVM and RFC was confirmed in the two external validation cohorts. In the Shanghai retrospective cohort (*N* = 1,465), SVM achieved the highest accuracy (83.82%), while in the Santai retrospective cohort (*N* = 714), RFC performed robustly (accuracy 81.51%) (Table 1).

**Table 1:** The overall predictive performance in the constitution types of TCM.

Model	Accuracy (%)	Kappa value	95% confidence interval (%)	Average sensitivity (%)	Mean specificity (%)	Average positive predictive value (%)	Average negative predictive value (%)
Internal validation *N* = 25,586							
SVM	99.47	0.991	99.26–99.63	87.93	99.88	97.91	99.92
RFC	95.48	0.9218	94.94–95.98	56.67	99.95	94.71	99.19
KNN	88.74	0.7989	87.94–89.50	28.59	99.95	88.09	98.85
NN	85.25	0.7171	84.36–86.11	22.67	96.54	73.63	97.82
DT	76.18	0.524	75.11–77.22	19.34	95.64	80.13	96.50
External validation Shanghai review queue *N* = 1,465							
SVM	83.82	0.7754	81.84–85.67	63.51	97.42	67.98	96.92
RFC	73.24	0.6152	70.90–75.49	35.14	98.25	63.91	94.87
KNN	64.71	0.4626	62.20–67.16	29.16	98.95	58.86	93.77
NN	59.32	0.3432	56.75–61.85	11.44	99.76	65.21	93.85
DT	51.54	0.1751	48.94–54.12	16.02	99.97	60.20	92.37
External validation Santai queues *N* = 714							
RFC	81.51	0.6786	78.47–84.30	42.57	96.18	39.12	96.35
SVM	80.95	0.682	77.88–83.77	46.85	96.51	41.94	96.66
KNN	77.17	0.564	73.91–80.20	33.12	96.85	36.01	96.19
NN	77.45	0.5292	74.21–80.47	24.55	97.32	45.94	96.12
DT	72.27	0.3465	68.83–75.52	19.98	97.81	41.04	95.28

RFC, Random Forest Classifier; DT, Decision Tree; SVM, Support Vector Machine; KNN, K-Nearest Neighbors; NN, Neural Network.

#### 3.1.2 Performance on specific constitution types

Given their strong overall performance, the strong performance of SVM and RFC holds potential for advancing from individual assessment to large-scale population-level constitution monitoring. Consequently, we further analyzed their performance on specific constitution types (detailed results in Table 2). In the internal validation, SVM was particularly precise, achieving prediction accuracies exceeding 98% for BC, QDC, YiDC, YaDC, PDC, and QSC. In comparison, RFC’s high accuracy (>95%) was limited to only BC and PDC, with significantly weaker performance on several other imbalanced constitution types.

**Table 2:** The perdiation accuracy of various models in the different constitution types of TCM.

Constitution type (%)	BC	QDC	YaDC	YiDC	PDC	DC	BSC	QSC	ISC
Internal validation *N* = 25,586									
SVM	99.94	98.77	96.41	98.49	99.84	88.46	40	100	70
RFC	99.39	67.49	82.64	84.17	96.94	38.46	0	20	20
KNN	96.76	44.79	58.08	59.3	91.16	7.69	0	0	0
NN	99.94	0	1.8	3.02	99.29	0	0	0	0
DT	97.54	0	0	0	73.38	0	0	0	0
External validation Shanghai review queue *N* = 1,465									
SVM	96.29	81.37	83.74	70.7	78.45	37.5	21.88	52.78	60
RFC	95.83	44.72	66.67	57.96	73.50	0	0	0	0
KNN	95.36	20.5	33.33	40.13	68.55	0	0	0	0
NN	97.37	0	0.81	1.27	83.39	0	0	0	0
DT	97.68	0	0	0	43.46	0	0	0	0
External validation Santai queues *N* = 714									
SVM	80.57	22.22	94.29	69.57	85.42	0	0	75	0
RFC	83.89	22.22	90	56.52	86.11	0	0	0	0
KNN	87.42	0	48.57	30.43	79.17	0	0	0	0
NN	92.27	0	1.43	8.7	91.67	0	0	0	0
DT	96.91	0	0	0	53.47	0	0	0	0

RFC, Random Forest Classifier; DT, Decision Tree; SVM, Support Vector Machine; KNN, K-Nearest Neighbors; NN, Neural Network; BC, Balanced constitution; QDC, Qi-deficiency constitution; YaDC, Yang-deficiency constitution; YiDC, Yin-deficiency constitution; PDC, Phlegm-dampness constitution; DHC, Damp-heat constitution; BSC, Blood-stasis constitution; QSC, Qi-stagnation constitution; ISC, Inherited-special constitution.

#### 3.1.3 External validation and generalization

In the external validation cohorts, the performance of both SVM and RFC showed a general decline. For instance, in the Shanghai cohort, their prediction accuracies for PDC dropped by over 20% (to 78.45 and 73.50%, respectively). Notably, however, both models performed exceptionally well for YaDC in the Santai cohort, with accuracies (94.29% for SVM, 90.00% for RFC) that approached or even surpassed the results from the internal validation. Conversely, neither model provided reliable predictive evidence for DHC, BSC, QSC, and SBC in the external validation sets.

### 3.2 RFC and SVM exhibit strong performance in predicting constitution transformation trend classification

This section evaluates the overall performance of the five machine learning models (SVM, RFC, KNN, NN, and DT) in predicting constitution transformation trend based on the 33 measurement items.

#### 3.2.1 Overall model performance

In the internal validation dataset (*N* = 25,586), RFC and SVM again demonstrated superior predictive performance, with RFC showing a significant advantage across all metrics. It achieved an accuracy of 96.92% (95% CI: 96.50–97.30%) and a Kappa value of 0.9505, indicating almost perfect agreement between the model’s predictions and the actual outcomes. Its average sensitivity (96.69%) and specificity (98.30%) were both excellent. The SVM model followed with an accuracy of 84.57% (95% CI: 83.72–85.39%) and a Kappa of 0.7536. Although its performance was notably inferior to RFC, its high specificity (91.65%) and substantial Kappa value suggest good discriminative ability in distinguishing different transformation trends. Notably, while the performance of NN, KNN, and DT was moderate, it represented a considerable improvement over their performance in the constitution type classification task. In external validation, the accuracies of RFC and SVM decreased to approximately 67–68%, yet both models maintained good specificities (82.13 and 82.27%, respectively), indicating robust stability in identifying negative cases (Table 3).

**Table 3:** The overall predictive performance in the constitution transformation trends of TCM.

Model	Accuracy (%)	Kappa value	95% confidence interval (%)	Average sensitivity (%)	Mean specificity (%)	Average positive predictive value (%)	Average negative predictive value (%)
Internal validation *N* = 25,586							
RFC	96.92	0.9505	96.50–97.30	96.69	98.3	96.82	98.34
SVM	84.57	0.7536	83.72–85.39	84.97	91.65	84.03	91.43
NN	81.5	0.6999	80.60–82.38	80.23	89.64	81.75	90.03
KNN	80.86	0.6891	79.95–81.76	77.26	89.93	78.4	90.47
DT	69.71	0.5041	68.64–70.75	68.62	82.56	70.75	83.56
External validation Santai queues *N* = 714							
RFC	67.41	0.4706	64.31–70.40	63.91	82.13	68.22	84.31
SVM	67.94	0.481	64.85–70.92	65.13	82.27	66.81	83.12
NN	63.68	0.419	60.52–66.77	60.52	80.71	61.78	80.95
KNN	56.66	0.2751	53.42–59.85	49.05	75.87	53.83	78.17
DT	68.16	0.479	65.07–71.13	64.82	81.97	68.65	83.07
External validation Shanghai review queue *N* = 1,465							
RFC	67.41	0.4706	64.31–70.40	63.91	82.13	68.22	84.31
SVM	67.94	0.481	64.85–70.92	65.13	82.27	66.81	83.12
NN	63.68	0.419	60.52–66.77	60.52	80.71	61.78	80.95
KNN	57.19	0.2853	53.95–60.38	49.86	76.31	55.03	78.41
DT	68.16	0.479	65.07–71.13	64.82	81.97	68.65	83.07

RFC, Random Forest Classifier; DT, Decision Tree; SVM, Support Vector Machine; KNN, K-Nearest Neighbors; NN, Neural Network.

#### 3.2.2 Performance in predicting constitution transformation trend

We further evaluated the models” performance in predicting each of the three specific transformation trends: “better,” “uncertain,” and “worse” (Table 4). In the internal validation, RFC maintained its leading performance, with high and stable prediction accuracies for the “better” (97.42%), “uncertain” (95.02%), and “worse” (97.64%) categories. In the external validation, however, the performance of all models declined, particularly for predicting the “worse” feature. In the Santai (*N* = 714) and Shanghai (*N* = 1,465) cohorts, the prediction accuracies of RFC and SVM for the “worse” feature dropped to as low as 20.00 and 44.00%, respectively.

**Table 4:** The predictive accuracy in the different transformation trends of TCM.

Model	Worse (%)	Uncertain (%)	Better (%)
Internal validation *N* = 25,586			
RFC	97.64	95.02	97.42
SVM	79.60	91.15	84.15
NN	69.42	85.11	86.17
KNN	68.19	72.04	91.55
DT	39.83	89.98	76.05
External validation Santai queues *N* = 714			
RFC	20.00	89.66	82.06
SVM	44.00	72.84	78.56
NN	44.00	62.50	75.05
KNN	24.40	40.09	82.71
DT	48.00	66.38	80.09
External validation Shanghai review queue *N* = 1,465			
RFC	20.00	89.66	82.06
SVM	44.00	72.84	78.56
NN	44.00	62.50	75.05
KNN	27.20	40.09	82.28
DT	48.00	66.38	80.09

RFC, Random Forest Classifier; DT, Decision Tree; SVM, Support Vector Machine; KNN, K-Nearest Neighbors; NN, Neural Network.

### 3.3 Predictive performance of biochemical indicators for constitution types and transformation trend

Modern TCM clinical practice frequently incorporates physiological and biochemical indicators for diagnosis. In this study, we selected 13 commonly used clinical biochemical indicators (Hb, WBC, PLT, FBG, ALT, AST, TBIL, SCr, BUN, TC, TG, LDL-C, and HDL-C) to evaluate their performance in predicting both constitution types and their transformation features.

#### 3.3.1 Performance in predicting constitution types

The five ML models were used to test the predictive performance of the 13 biochemical indicators for constitution types (Table 5). The results showed that RFC, SVM, and DT achieved comparable overall accuracies of approximately 70% (70.39, 70.25, and 70.53%, respectively). While the average sensitivity of the models was generally low (ranging from 17.71 to 21.54%), their specificities were high, with all models exceeding 80% and RFC reaching up to 93.20%. In predictions for specific constitution types (Table 6), the models performed well only for BC and PDC. For instance, the accuracies of RFC, SVM, and NN for predicting BC were 87.38, 85.28, and 99.97%, respectively. For PDC, most models (excluding NN) achieved accuracies above 65%, which may be attributed to the lipid metabolism abnormalities commonly associated with this constitution. However, the poor performance for other constitution types suggests a weaker association with these 13 selected indicators.

**Table 5:** The overall performance of each model based on biochemical indicators.

Model	Accuracy (%)	Kappa value	95% confidence interval (%)	Average sensitivity (%)	Mean specificity (%)	Average positive predictive value (%)	Average negative predictive value (%)
Constitution types							
RFC	70.39	0.4387	69.25–71.51	17.71	93.20	40.57	91.61
SVM	70.25	0.4515	69.11–71.37	21.54	89.41	45.73	91.60
NNk	56.53	0.0008	55.30–57.75	11.14	83.33	27.37	87.34
KNN	53.70	0.0684	52.47–54.93	11.55	89.66	17.30	87.76
DT	70.53	0.444	69.40–71.65	20.36	89.36	47.53	91.60
Constitution transformation trend							
RFC	72.92	0.4823	71.81–74.00	58.01	81.60	59.28	84.38
SVM	72.85	0.4832	71.75–73.94	58.18	81.66	72.58	84.34
NN	72.73	0.4804	71.62–73.82	58.02	81.58	72.43	84.25
KNN	54.61	0.0621	53.38–55.83	36.23	68.33	38.18	69.85
DT	72.85	0.4832	71.75–73.94	58.18	81.66	72.58	84.34

RFC, Random Forest Classifier; DT, Decision Tree; SVM, Support Vector Machine; KNN, K-Nearest Neighbors; NN, Neural Network.

**Table 6:** The predictive accuracy in the different consititution types of TCM based on biochemical index modeling.

Model (%)	Constitution type								
	BC	QDC	YaDC	YiDC	PDC	DH	BSC	QSC	ISC
RFC	87.38	2.45	0.60	19.10	68.74	0	0	0	0
SVM	85.28	0	0	34.67	69.34	0	0	0	0
NN	99.97	0	0.30	0	0	0	0	0	0
KNN	81.16	0	0.60	0.25	27.11	0	0	0	0
DT	87.11	0	0	22.61	69.34	0	0	0	0

RFC, Random Forest Classifier; DT, Decision Tree; SVM, Support Vector Machine; KNN, K-Nearest Neighbors; NN, Neural Network; BC, Balanced constitution; QDC, Qi-deficiency constitution; YaDC, Yang-deficiency constitution; YiDC, Yin-deficiency constitution; PDC, Phlegm-dampness constitution; DHC, Damp-heat constitution; BSC, Blood-stasis constitution; QSC, Qi-stagnation constitution; ISC, Inherited-special constitution.

#### 3.3.2 Performance in predicting constitution transformation trend

The models’ performance improved significantly when predicting constitution transformation trends. With the exception of KNN, the other four models achieved accuracies exceeding 70%, with average sensitivity notably increasing to approximately 58% while specificity remained stable at around 81% (Table 6). This suggests that the dynamic changes in biochemical indicators are more strongly correlated with the process of constitutional transformation. As shown in Table 7, when predicting specific trends, all five models achieved accuracies over 80% for the “better” state. For the “uncertain” state, four models (excluding KNN) reached accuracies approaching or exceeding 90%. In stark contrast, all models performed extremely poorly in predicting the “worse” state, with the highest accuracy being only 4.01%. These findings indicate that trends of normalization (“better”) and fluctuation (“uncertain”) in biochemical markers are more readily captured by the models, whereas the “worse” trend may involve more complex pathophysiological mechanisms that require a broader range of indicators for effective prediction.

**Table 7:** The predictive accuracy in the different transformation trends of TCM based on biochemical index modeling.

Model (%)	TCM transformation trend		
	Class: Go bad	Class: Uncertain	Class: Get better
RFC	0.10	89.62	84.31
SVM	0.00	90.91	83.64
NN	0.00	90.39	83.67
KNN	4.01	21.63	82.99
DT	0.00	90.91	83.64

RFC, Random Forest Classifier; DT, Decision Tree; SVM, Support Vector Machine; KNN, K-Nearest Neighbors; NN, Neural Network.

## 4 Discussion

### 4.1 Comparing algorithms to delineate decision boundaries at follow-up time points

Dynamic constitution assessment requires classifying the current state at each follow-up while capturing high-dimensional, nonlinear co-variation among symptom clusters (16). Algorithm comparison clarifies which models best fit these properties. SVM achieved 99.47% internal accuracy (see Table 2), leveraging kernel methods (e.g., RBF) to separate complex boundaries, with particularly strong performance when features are clearly demarcated and clinically specific (e.g., BC, PDC). RFC, through multi-tree ensembles and Gini-based importance, identified core symptom combinations and remained robust for morpho-metabolic patterns such as PDC. Both models showed limitations for types relying on micro-level signs and weak cross-dimensional signals (e.g., BSC), indicating measurement blind spots in current instruments that warrant finer objective indices and standardized observational items. Overall, SVM is effective for delineating complex nonlinear boundaries at a given time point, whereas RFC emphasizes stable feature combinations and robustness (17).

### 4.2 From 81 transformation events to three directional trends (“better–uncertain–worse”)

Collapsing 81 semantically similar yet sparse transformation events into three directional categories provides a structured representation of transformation, aligns with clinical semantics (“improved/indeterminate/worsened”), reduces class fragmentation and labeling noise, increases per-class effective sample size, and improves learnability and transportability over time (18). This 3-category framework is a methodological trade-off: it simplifies a sparse and statistically unstable 81-class problem into a high-level, clinically actionable signal aligned with the “Zhi Wei Bing” goal (i.e., “moving toward or away from” the balanced state), but it does so at the cost of losing granular detail about specific nuanced transitions. Model performance changed in a principled way after reframing: RFC accuracy increased to 96.92% in the trend task (about +12.44% versus type classification, see Table 3), indicating that its ensemble mechanism captures coordinated and gradual multi-dimensional changes; SVM reached 84.57%, lower than in time-point classification, reflecting its preference for sharp decision boundaries over directional inference. In external validation, predictions for “worse” deteriorated markedly (RFC from 97.64% internally to about 20%, SVM to about 44%, see Table 4), consistent with high real-world heterogeneity, class imbalance, limited sample size, and temporal non-stationarity; the “worse” state often reflects fluctuating, layered pathological burdens that are difficult to represent with a limited feature set. Overall, the three-category scheme is clinically and methodologically sound, improves stability and usability, yet “worse” requires larger samples, longer follow-up, richer lifestyle and exposure data, objective signs, and explicit temporal feature engineering.

#### 4.2.1 Clinical implementation strategy

Integrating this framework into the ‘Zhi Wei Bing’ paradigm involves a proactive workflow. In community health centers, once an elderly individual completes the TCMECQ, the model automatically predicts their future constitutional trajectory. A prediction of a ‘Worse’ trend serves as a red-flag alert, prioritizing the individual for immediate TCM intervention—such as specific dietary guidance or herbal conditioning—before overt pathology manifests. This shifts health management from passive treatment to active prevention, optimizing resource allocation for high-risk groups.

### 4.3 Rationale for the 13 biochemical indicators, TCM relevance, and predictive role

To support scalable follow-up and checkup scenarios and to cover multiple physiological systems, we included 13 routine, accessible, and reproducible indicators (Hb, WBC, PLT, FBG, ALT, AST, TBIL, SCr, BUN, TC, TG, LDL-C, and HDL-C). These span hematologic/inflammatory status, glyco-lipid metabolism, hepatobiliary function and bilirubin metabolism, and renal/uremic function, corresponding to TCM dimensions of Blood (19), phlegm-dampness/damp-heat (20), liver-gallbladder regulation (21), and kidney essence/qi (22, 23). For time-point classification based on these indicators, overall accuracy was about 70% with low sensitivity and high specificity (Table 7); models identified BC and PDC relatively well, consistent with known links between PDC and lipid dysregulation. RFC explained about 68.74% of variance for PDC but ≤2.45% for QDC, suggesting stronger correspondence for metabolic phenotypes than for function-dominant types. For trend prediction, except for KNN, all models exceeded 70% accuracy with mean sensitivity around 58% and specificity around 81% (see Table 5). The “better” state was well identified (overall about 84.31%), demonstrating practical cross-marker synergy. External evidence for “worse” remained weak, indicating that these 13 indicators alone are insufficient to stably characterize deteriorating trajectories; richer metabolic and inflammatory profiles, objective signs, denser longitudinal sampling, and imbalance-aware learning strategies are needed. Overall, biochemical indicators more readily support recognition of ‘better/uncertain,’ whereas their failure to predict the ‘Worse’ trend (Accuracy < 5%) offers a critical insight: TCM constitution likely represents a functional, holistic physiological state that is orthogonal to the structural pathologies measured by routine blood tests. The deterioration of constitution involves complex, multi-system functional changes that cannot be captured by single biochemical markers alone. This effectively validates the independent clinical value of the TCMECQ, as it captures health information that standard biochemical panels miss, highlighting the necessity of multi-modal assessment in future research.

## 5 Strengths and limitations

In summary, this study reveals the potential of ML methods in predicting TCM constitution types and their transformation trends, establishing for the first time a ML framework for TCM constitution transformation. Its primary strengths are twofold. Firstly, through task reframing, we consolidated 81 complex transformation events into three intuitive trends (“better,” “uncertain,” “worse”). This approach effectively fuses the holistic perspective of TCM with the principles of machine learning feature engineering and significantly enhanced the capability of ensemble models like RFC to capture dynamic trends. Secondly, by systematically comparing different algorithms, the study revealed the dynamic synergistic patterns between morpho-anatomical features and functional indicators, highlighting the superiority of SVM for point-in-time classification with clear boundaries and the robustness of RFC for predicting trends based on synergistic symptom cluster changes. Finally, our research creates a preliminary quantitative link between modern biochemical indicators and TCM constitution theory, providing empirical evidence for the strong association between combined marker changes (e.g., a synergistic decrease in FBG and ALT) and the “better” constitutional trend.

However, several limitations must be acknowledged. The most critical limitation is the poor generalizability and evidence of overfitting. As shown in our results, there was a stark performance drop between the high internal validation accuracy (e.g., 97.64% for “worse” trend) and the poor external validation accuracy (as low as 20% for the “worse” trend). This highlights a significant challenge in generalizing the model to new populations, likely due to regional, lifestyle, and environmental heterogeneity, and indicates overfitting in the current model.

Furthermore, regarding international adaptability, it is important to note that TCM constitution is a universal life phenomenon, not limited to a specific ethnicity. Assessment scales for TCM constitution have been validated in multiple countries (e.g., United States, Japan, South Korea), indicating that the constitutional types and the phenomenon of dynamic transformation are widely present across different populations. Therefore, the machine learning framework and the transformation patterns identified in this study have potential global applicability. However, since our model was trained on a specific cohort in Southwest China, and constitutional characteristics can be influenced by geography, diet, and genetics, the direct application of this specific model to other countries may yield variations. Future international applications would benefit from a “transfer learning” strategy—using our framework as a base and fine-tuning it with local population data to ensure precision.

Second, this study did not mitigate the significant “real-world” data imbalance. This resulted in poor predictive performance for rare constitution types (e.g., DHC, BSC). We consciously chose not to use artificial oversampling (like SMOTE) to avoid distorting the epidemiological reality, but this trade-off means the current model’s utility is high for common constitutions but low for rare ones.

Third, the models remain “black boxes”. The lack of interpretability analysis (e.g., using SHAP or LIME) is a major barrier to clinical trust and utility, limiting our understanding of why the model makes a prediction.

Finally, other data and feature limitations persist: the sample size of the prospective cohort was relatively insufficient, which may have increased errors in trend prediction; the study did not incorporate emerging biomarkers such as epigenetic data, potentially limiting the model’s comprehensiveness and accuracy; and the constitution scale itself has measurement blind spots in its micro-diagnostic dimensions.

Future research could focus on three key areas: (1) constructing multi-modal deep learning networks that integrate multi-omics, imaging, and objective signs to enhance predictive accuracy and comprehensiveness, while employing interpretability tools (e.g., SHAP) to demystify model decision-making; (2) developing dynamic intervention recommendation systems based on transformation patterns; and (3) expanding this comparative framework to include other advanced ensemble and deep learning approaches (e.g., XGBoost, Adaboost, and CNN-based models) to assess generalizability and performance consistency across multi-center datasets, incorporating cross-validation benchmarks from international studies to enhance methodological transparency and comparability.

## Data Availability

The original contributions presented in the study are included in the article/Supplementary material, further inquiries can be directed to the corresponding author.

## References

[1] Li S Zhu P Cai G Li J Huang T Tang W. Application of machine learning models in predicting insomnia severity: an integrative approach with constitution of traditional Chinese medicine. Front Med (Lausanne). (2023) 10:1292761. doi: 10.3389/fmed.2023.1292761, 37928471 PMC10625410

[2] Bai MH Wang J Zheng YF Li YS Hou SJ Li LR . Analysis of the distribution characteristics of traditional Chinese medicine constitution types in the Chinese population based on 108015 sample data. J Beijing Univ Tradit Chin Med. (2020) 43:498–507.

[3] Su SY Yang CH Chiu CC Wang Q. Acoustic features for identifying constitutions in traditional Chinese medicine. J Altern Complement Med. (2013) 19:569–76. doi: 10.1089/acm.2012.0478, 23270320

[4] Zhang J Hou S Wang J Li L Li P Han J . Classification of traditional Chinese medicine constitution based on facial features in color images. J Tradit Chin Med Sci. (2016) 3:141–6. doi: 10.1016/j.jtcms.2016.12.001

[5] Wang YC Bai LN. Classification of body constitution of pulse signal in TCM based on BP neural network. J Tradit Chin Med. (2014) 55:11.

[6] Huan EY Wen GH Zhang SJ Li DY Hu Y Chang TY . Deep convolutional neural networks for classifying body constitution based on face image. Comput Math Methods Med. (2017) 2017:9846707. doi: 10.1155/2017/9846707, 29181087 PMC5664380

[7] Wen G Ma J Hu Y Li H Jiang L. Grouping attributes zero-shot learning for tongue constitution recognition. Artif Intell Med. (2020) 109:101951. doi: 10.1016/j.artmed.2020.101951 34756217

[8] TCM. Classification and determination of constitution in traditional Chinese medicine (ZYYXH/T157-2009). World J Integr Tradit Western Med. (2009) 4:303–4.

[9] Wong W Lam CL Su YC Lin SJ Ziea ET Wong VT . Measuring body constitution: validation of the body constitution questionnaire (BCQ) in Hong Kong. Complement Ther Med. (2014) 22:670–82. doi: 10.1016/j.ctim.2014.05.009, 25146072

[10] Wang Q. Using complex system science thinking to decode life sciences in traditional Chinese medicine constitution. J Beijing Univ Tradit Chin Med. (2023) 46:889–96.

[11] Aydın S. Cross-validated Adaboost classification of emotion regulation strategies identified by spectral coherence in resting-state. Neuroinformatics. (2022) 20:627–39. doi: 10.1007/s12021-021-09542-7 34536200

[12] Aydın S Demirtaş S Yetkin S. Cortical correlations in wavelet domain for estimation of emotional dysfunctions, neural computing and applications (2018) 30:1085–94.

[13] Aydın S Akın B. Machine learning classification of maladaptive rumination and cognitive distraction in terms of frequency specific complexity, biomedical signal processing and control (2022) 77:103740. doi: 10.1016/j.bspc.2022.103740,

[14] Wang Q. Three treatises on constitution in traditional Chinese medicine. J Beijing Univ Tradit Chin Med. (2008) 1:653–5.

[15] Liu BY Wang YY. A study on the concepts and relationships of syndrome, pattern, and symptom. J Tradit Chin Med. (2007) 1:293–6+298. doi: 10.13288/j.11-2166/r.2007.04.001

[16] Sun W Bai M Wang J Wang B Liu Y Wang Q . Machine learning-assisted rapid determination for traditional Chinese medicine constitution. Chin Med. (2024) 19:127. doi: 10.1186/s13020-024-00992-0, 39278905 PMC11403957

[17] Li J Zhai Y Cao Y Xia Y Yu R. Development of an interpretable machine learning model associated with genetic indicators to identify yin-deficiency constitution. Chin Med. (2024) 19:71. doi: 10.1186/s13020-024-00941-x, 38750482 PMC11094929

[18] Hu J Fung FW Jacobwitz M Parikh DS Vala L Donnelly M . Machine learning models to predict electroencephalographic seizures in critically ill children. Seizure. (2021) 87:61–8. doi: 10.1016/j.seizure.2021.03.001, 33714840 PMC8044039

[19] Liang X Wang Q Jiang Z Li Z Zhang M Yang P . Clinical research linking traditional Chinese medicine constitution types with diseases: a literature review of 1639 observational studies. J Tradit Chin Med. (2020) 40:690–702. doi: 10.19852/j.cnki.jtcm.2020.04.019, 32744037

[20] Yu R Liu D Yang Y Han Y Li L Zheng L . Expression profiling-based clustering of healthy subjects recapitulates classifications defined by clinical observation in Chinese medicine. J Genet Genomics. (2017) 44:191–7. doi: 10.1016/j.jgg.2017.01.001, 28412226

[21] Liu Y Wang M Luo Y Chen J Lu Y Shi Y . MiRNA-target network analysis identifies potential biomarkers for traditional Chinese medicine (TCM) syndrome development evaluation in hepatitis B caused liver cirrhosis. Sci Rep. (2017) 7:11054. doi: 10.1038/s41598-017-11351-5, 28887510 PMC5591282

[22] Wang SJ Yue W Rahman K Xin HL Zhang QY Qin LP . Mechanism of treatment of kidney deficiency and osteoporosis is similar by traditional Chinese medicine. Curr Pharm Des. (2016) 22:312–20. doi: 10.2174/1381612822666151112150346, 26561071

[23] Gan JW Lv DX Fu J Shi LY Yuan CY Zeng XQ . Effectiveness of Zhenqi Buxue Oral liquid combined with progesterone for treatment of Oligomenorrhea and Hypomenorrhea with qi-blood and kidney (Shen) essence deficiency: a randomized controlled trial. Chin J Integr Med. (2023) 29:963–70. doi: 10.1007/s11655-023-3740-y, 37594704

