# Supplementary material for: Application of machine learning methods in prediction of the body constitution types and transformation trends of traditional Chinese medicine: from the datasets of questionnaire survey on elderly people in Southwest China
Source: Front Med (Lausanne). 2026 Jan 15;13:1698576. doi: 10.3389/fmed.2026.1698576 (PMC12852432; doi:10.3389/fmed.2026.1698576)
Supplement: Supplementary file 1 [file Table_1.DOCX]

**Supplementary Table 1** The charactistics of 9 basic types of body constitutions

| Constitution Type | Type charactistic |
| --- | --- |
| BC | Harmonious balance of Yin and Yang, moderate body state, reddish complexion, and energetic spirit are the main characteristics |
| QDC | Deficiency of Yuan Qi, manifested by fatigue, shortness of breath, and spontaneous sweating as the main features |
| YaDC | Characterized by a deficiency of Yang Qi, leading to intolerance to cold, cold limbs, and other manifestations of internal cold due to weakened physiological function. |
| YiDC | Characterized by a deficiency of Yin fluids, resulting in signs of internal heat such as dry mouth and throat, warm palms and soles, and restlessness. |
| PDC | Caused by accumulation of phlegm and dampness due to spleen dysfunction, with features such as heaviness in the body, chest oppression, and greasy tongue coating. |
| DHC | Marked by the combination of internal dampness and heat, often showing oily skin, bitter taste in the mouth, yellow greasy tongue coating, and irritability. |
| BSC | Arises from impeded blood circulation, presenting with dark or purplish complexion, localized pain, and tendency toward bruising or blood stasis. |
| QSC | Results from stagnation of Qi movement, with emotional depression, frequent sighing, chest and flank tightness, and mood instability. |
| ISC | Due to congenital abnormalities or inherited factors, characterized by hypersensitivity (e.g. allergies), abnormal responses, and susceptibility to certain conditions. |

BC: Balanced constitution, QDC: Qi-deficiency constitution, YaDC: Yang-deficiency constitution, YiDC: Yin-deficiency constitution, PDC: Phlegm-dampness constitution, DHC: Damp-heat constitution, BSC: Blood-stasis constitution, QSC: Qi-stagnation constitution, ISC: Inherited-special constitution.
