# Supplementary material for: Application of machine learning methods in prediction of the body constitution types and transformation trends of traditional Chinese medicine: from the datasets of questionnaire survey on elderly people in Southwest China
Source: Front Med (Lausanne). 2026 Jan 15;13:1698576. doi: 10.3389/fmed.2026.1698576 (PMC12852432; doi:10.3389/fmed.2026.1698576)
Supplement: Supplementary file 2 [file Table_2.DOCX]

**Supplementary Table 2** Transformation trends of 81 Constitution Transition Events of TCM

| before | after | | | | | | | | |
| --- | --- | --- | --- | --- | --- | --- | --- | --- | --- |
|  | BC | QDC | YaDC | YiDC | PDC | DHC | BSC | QSC | ISC |
| BC | + | - | - | - | - | - | - | - | - |
| QDC | + | ？ | - | - | - | ? | - | - | ? |
| YaDC | + | + | ？ | ? | - | ? | - | - | ? |
| YiDC | + | + | ？ | ？ | ? | - | - | - | ? |
| PDC | + | + | + | ? | ？ | ? | - | - | ? |
| DHC | + | ？ | ？ | + | ？ | ? | - | - | ? |
| BSC | + | + | + | + | + | + | ? | + | ? |
| QSC | + | + | + | + | + | + | - | ? | ? |
| ISC | + | ？ | ？ | ? | ? | ? | ? | ? | ? |

BC: Balanced constitution, QDC: Qi-deficiency constitution, YaDC: Yang-deficiency constitution, YiDC: Yin-deficiency constitution, PDC: Phlegm-dampness constitution, DHC: Damp-heat constitution, BSC: Blood-stasis constitution, QSC: Qi-stagnation constitution, ISC: Inherited-special constitution, +: better , ?: uncertain, -: worse.
