# Supplementary material for: Application of machine learning methods in prediction of the body constitution types and transformation trends of traditional Chinese medicine: from the datasets of questionnaire survey on elderly people in Southwest China
Source: Front Med (Lausanne). 2026 Jan 15;13:1698576. doi: 10.3389/fmed.2026.1698576 (PMC12852432; doi:10.3389/fmed.2026.1698576)
Supplement: Supplementary file 4 [file Table_4.DOCX]

| Evaluation Table for Transition Trends of Elderly Constitutions in Traditional Chinese Medicine | | | | | | | | | |
| --- | --- | --- | --- | --- | --- | --- | --- | --- | --- |
| Dear Expert, | | | | | | | | | |
| Hello! | | | | | | | | | |
| We are conducting a survey on the constitution of the elderly in Traditional Chinese Medicine. We kindly ask you to evaluate the transition trends of elderly individuals' constitutions. Each transition trend is assigned a value of "↑," defining this transition as favorable. For example, a transition from "Qi Deficiency Constitution" to "Balanced Constitution" is considered favorable. If you agree, please fill in "1"; if you are uncertain, please fill in "0"; and if you disagree, please fill in "-1." Please note that if you fill in "-1," we will interpret it as your belief that this transition state is unfavorable. | | | | | | | | | |
| Thank you for your support! | | | | | | | | | |
|  | | | | | | | | | |
| Before | After | | | | | | | | |
|  | Balanced constitution | Inherited-special constitution | Blood-stasis constitution | Qi-stagnation constitution | Damp-heat constitution | Phlegm-dampness constitution | Yin-deficiency constitution | Yang-deficiency constitution | Qi-deficiency constitution |
| Qi-deficiency constitution | ↑ | ↑ | ↑ | ↑ | ↑ | ↑ | ↑ | ↑ | ↑ |
| Yang-deficiency constitution | ↑ | ↑ | ↑ | ↑ | ↑ | ↑ | ↑ | ↑ |  |
| Yin-deficiency constitution | ↑ | ↑ | ↑ | ↑ | ↑ | ↑ | ↑ |  |  |
| Phlegm-dampness constitution | ↑ | ↑ | ↑ | ↑ | ↑ | ↑ |  |  |  |
| Damp-heat constitution | ↑ | ↑ | ↑ | ↑ | ↑ |  |  |  |  |
| Qi-stagnation constitution | ↑ | ↑ | ↑ | ↑ |  |  |  |  |  |
| Blood-stasis constitution | ↑ | ↑ | ↑ |  |  |  |  |  |  |
| Inherited-special constitution | ↑ | ↑ |  |  |  |  |  |  |  |
| Balanced constitution | ↑ |  |  |  |  |  |  |  |  |
